# 4-{2-[2-(4-Chloro­benzyl­idene)hydrazinyl­idene]-3,6-dihydro-2*H*-1,3,4-thia­diazin-5-yl}-3-phenyl­sydnone

**DOI:** 10.1107/S1600536811013912

**Published:** 2011-04-22

**Authors:** Hoong-Kun Fun, Wan-Sin Loh, Nithinchandra, Balakrishna Kalluraya

**Affiliations:** a X-ray Crystallography Unit, School of Physics, Universiti Sains Malaysia, 11800 USM, Penang, Malaysia; b Department of Studies in Chemistry, Mangalore University, Mangalagangotri, Mangalore 574 199, India

## Abstract

The title compound, C_18_H_13_ClN_6_O_2_S, exists in *trans* and *cis* configurations with respect to the acyclic C=N bonds [C=N = 1.2837 (15) and 1.3000 (14) Å, respectively]. The 3,6-dihydro-2*H*-1,3,4-thia­diazine ring adopts a half-boat conformation. The sydnone ring is approximately planar [maximum deviation = 0.002 (1) Å] and forms dihedral angles of 50.45 (7) and 61.21 (6)° with the aromatic rings. In the crystal, inter­molecular N—H⋯N, C—H⋯Cl and C—H⋯S hydrogen bonds link the mol­ecules into layers parallel to *ab* plane. The crystal packing is stabilized by C—H⋯π inter­actions and further consolidated by π–π inter­actions involving the phenyl rings [centroid–centroid distance = 3.6306 (7) Å].

## Related literature

For background to sydnones and their biological activity, see: Newton & Ramsden (1982 ▶); Wagner & Hill (1974 ▶); Kalluraya & Rahiman (1997 ▶); Kalluraya *et al.* (2003 ▶). For related structures, see: Fun *et al.* (2010 ▶); Fun, Loh *et al.* (2011 ▶); Fun, Quah *et al.* (2011 ▶). For ring conformations, see: Cremer & Pople (1975 ▶). For bond-length data, see: Allen *et al.* (1987 ▶). For hydrogen-bond motifs, see: Bernstein *et al.* (1995 ▶). For the stability of the temperature controller used in the data collection, see: Cosier & Glazer (1986 ▶).
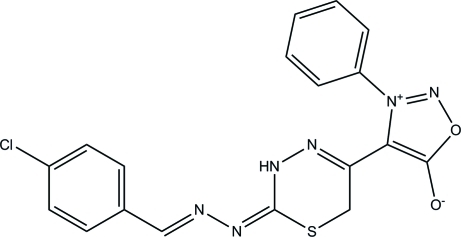


## Experimental

### 

#### Crystal data

C_18_H_13_ClN_6_O_2_S
*M* *_r_* = 412.85
Triclinic, 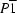

*a* = 7.3180 (3) Å
*b* = 10.1567 (5) Å
*c* = 12.4721 (6) Å
α = 96.686 (1)°
β = 95.285 (1)°
γ = 95.229 (1)°
*V* = 911.92 (7) Å^3^
*Z* = 2
Mo *K*α radiation
μ = 0.35 mm^−1^
*T* = 100 K
0.51 × 0.23 × 0.07 mm

#### Data collection

Bruker SMART APEXII DUO CCD area-detector diffractometer
Absorption correction: multi-scan (*SADABS*; Bruker, 2009 ▶) *T*_min_ = 0.842, *T*_max_ = 0.976
18115 measured reflections
6480 independent reflections
5506 reflections with *I* > 2σ(*I*)
*R*_int_ = 0.022

#### Refinement

*R*[*F* ^2^ > 2σ(*F* ^2^)] = 0.039
*wR*(*F* ^2^) = 0.113
*S* = 1.05
6480 reflections
257 parameters
H atoms treated by a mixture of independent and constrained refinement
Δρ_max_ = 0.53 e Å^−3^
Δρ_min_ = −0.27 e Å^−3^

### 

Data collection: *APEX2* (Bruker, 2009 ▶); cell refinement: *SAINT* (Bruker, 2009 ▶); data reduction: *SAINT*; program(s) used to solve structure: *SHELXTL* (Sheldrick, 2008 ▶); program(s) used to refine structure: *SHELXTL*; molecular graphics: *SHELXTL*; software used to prepare material for publication: *SHELXTL* and *PLATON* (Spek, 2009 ▶).

## Supplementary Material

Crystal structure: contains datablocks global, I. DOI: 10.1107/S1600536811013912/bq2294sup1.cif

Structure factors: contains datablocks I. DOI: 10.1107/S1600536811013912/bq2294Isup2.hkl

Additional supplementary materials:  crystallographic information; 3D view; checkCIF report

## Figures and Tables

**Table 1:** Hydrogen-bond geometry (Å, °) *Cg*2 is the centroid of the C1–C6 benzene ring.

*D*—H⋯*A*	*D*—H	H⋯*A*	*D*⋯*A*	*D*—H⋯*A*
N3—H1*N*3⋯N2^i^	0.84 (2)	2.03 (2)	2.8752 (14)	178 (2)
C9—H9*A*⋯Cl1^ii^	0.97	2.78	3.4904 (13)	130
C18—H18*A*⋯S1^iii^	0.93	2.86	3.6729 (12)	147
C17—H17*A*⋯*Cg*2^iv^	0.93	2.64	3.5208 (15)	158

Symmetry codes: (i) 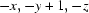; (ii) 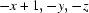; (iii) 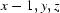; (iv) 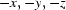.

**Fig. 1.**
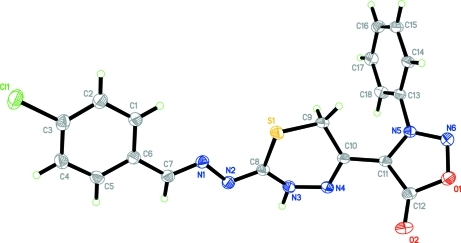
The molecular structure of the title compound, showing 50% probability displacement ellipsoids and the atom-numbering scheme.

**Fig. 2.**
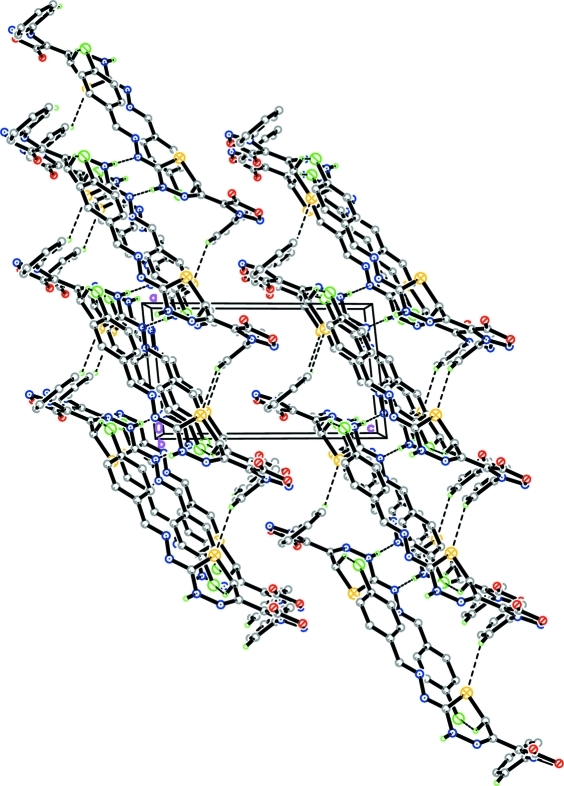
The crystal packing of the title compound, viewed along the b axis. H atoms not involved in the intermolecular interactions (dashed lines) have been omitted for clarity.

## supplementary crystallographic information

### Comment

Sydnones are a class of mesoionic compounds containing a 1,2,3-oxadiazole ring system. A number of sydnone derivatives have shown diverse biological activities such as anti-inflammatory, analgesic and anti-arthritic (Newton & Ramsden, 1982; Wagner & Hill, 1974) properties. Sydnones possessing heterocyclic moieties at the 4-position are also known for a wide range of biological properties (Kalluraya & Rahiman, 1997). Encouraged by these reports and in continuation of our research for biologically active nitrogen-containing heterocycles, a thiadiazine moiety at the 4-position of the phenylsydnone was introduced. The title compound was synthesized by the condensation of 4-bromoacetyl-3-arylsydnones with *N*'-[(4-chlorophenyl)methylidene]thiocarbonohydrazide. 4-Bromoacetyl-3-arylsydnones were in turn obtained by the photochemical bromination of 4-acetyl-3-arylsydnones (Kalluraya *et al.*, 2003).

The title compound (Fig. 1) exists in *trans* and *cis* configurations with respect to the acyclic C7═N1 and C8═N2 bonds [C7═N1 = 1.2837 (15) Å and C8═N2 = 1.3000 (14) Å], respectively. The 3,6-dihydro-2*H*-1,3,4-thiadiazine ring (N3/N4/C10/C9/S1) adopts a half-boat conformation with the puckering parameter (Cremer & Pople, 1975), Q = 0.5266 (11) Å; Θ = 108.31 (12)°; φ = 138.02 (13)°. The sydnone ring (N5/N6/O1/C12/C11) is approximately planar with a maximum deviation of 0.002 (1) Å at atom N5 and forms dihedral angles of 50.45 (7)° and 61.21 (6)° with the phenyl rings (C1–C6 & C13–C18), respectively. Bond lengths (Allen *et al.*, 1987) and angles are within normal ranges and are comparable to the related structures (Fun *et al.*, 2010; Fun & Loh *et al.*, 2011; Fun & Quah *et al.*, 2011).

In the crystal packing (Fig. 2), intermolecular N3—H1N3···N2, C9—H9A···Cl1 and C18—H18A···S1 hydrogen bonds (Table 1) link the molecules into layers parallel to *ab* plane. The crystal packing is stabilized by C—H···π interactions (Table 1) and further consolidated by π–π interactions (Table 1), involving the centroids of phenyl rings (*Cg*1; C13–C18) with the separation of *Cg*1···*Cg*1^v^ being 3.6306 (7) Å [symmetry code: (v) -1 - *x*, -*y*, 1 - *z*].

### Experimental

To a solution of 4-bromoacetyl-3-(*p*-anisyl)sydnone (0.01 mol) and *N*'-[(4-chlorophenyl)methylidene]thiocarbonohydrazide (0.01 mol) in ethanol, a catalytic amount of anhydrous sodium acetate was added. The solution was stirred at room temperature for 2–3 h. The solid product that separated out was filtered and dried. It was then recrystallized from ethanol. Crystals suitable for X-ray analysis were obtained from 1:2 mixtures of DMF and ethanol by slow evaporation.

### Refinement

H1N3 was located from the difference Fourier map and refined freely [N–H = 0.84 (2) Å]. The remaining H atoms were positioned geometrically and refined using a riding model with *U*_iso_(H) = 1.2 *U*_eq_(C) [C–H = 0.93 or 0.97 Å].

### Crystal data

**Table:**

C_18_H_13_ClN_6_O_2_S	*Z* = 2
*M**_r_* = 412.85	*F*(000) = 424
Triclinic, *P*1	*D*_x_ = 1.504 Mg m^−^^3^
Hall symbol: -P 1	Mo *K*α radiation, λ = 0.71073 Å
*a* = 7.3180 (3) Å	Cell parameters from 8568 reflections
*b* = 10.1567 (5) Å	θ = 2.8–35.0°
*c* = 12.4721 (6) Å	µ = 0.35 mm^−^^1^
α = 96.686 (1)°	*T* = 100 K
β = 95.285 (1)°	Plate, light purple
γ = 95.229 (1)°	0.51 × 0.23 × 0.07 mm
*V* = 911.92 (7) Å^3^	

### Data collection

**Table:**

Bruker SMART APEXII DUO CCD area-detector diffractometer	6480 independent reflections
Radiation source: fine-focus sealed tube	5506 reflections with *I* > 2σ(*I*)
graphite	*R*_int_ = 0.022
φ and ω scans	θ_max_ = 32.5°, θ_min_ = 2.8°
Absorption correction: multi-scan (*SADABS*; Bruker, 2009)	*h* = −11→11
*T*_min_ = 0.842, *T*_max_ = 0.976	*k* = −15→15
18115 measured reflections	*l* = −18→18

### Refinement

**Table:**

Refinement on *F*^2^	Primary atom site location: structure-invariant direct methods
Least-squares matrix: full	Secondary atom site location: difference Fourier map
*R*[*F*^2^ > 2σ(*F*^2^)] = 0.039	Hydrogen site location: inferred from neighbouring sites
*wR*(*F*^2^) = 0.113	H atoms treated by a mixture of independent and constrained refinement
*S* = 1.05	*w* = 1/[σ^2^(*F*_o_^2^) + (0.0611*P*)^2^ + 0.3032*P*] where *P* = (*F*_o_^2^ + 2*F*_c_^2^)/3
6480 reflections	(Δ/σ)_max_ = 0.001
257 parameters	Δρ_max_ = 0.53 e Å^−^^3^
0 restraints	Δρ_min_ = −0.27 e Å^−^^3^

### Special details

**Table:**

Experimental. The crystal was placed in the cold stream of an Oxford Cryosystems Cobra open-flow nitrogen cryostat (Cosier & Glazer, 1986) operating at 100.0 (1) K.
Geometry. All e.s.d.'s (except the e.s.d. in the dihedral angle between two l.s. planes) are estimated using the full covariance matrix. The cell e.s.d.'s are taken into account individually in the estimation of e.s.d.'s in distances, angles and torsion angles; correlations between e.s.d.'s in cell parameters are only used when they are defined by crystal symmetry. An approximate (isotropic) treatment of cell e.s.d.'s is used for estimating e.s.d.'s involving l.s. planes.
Refinement. Refinement of *F*^2^ against ALL reflections. The weighted *R*-factor *wR* and goodness of fit *S* are based on *F*^2^, conventional *R*-factors *R* are based on *F*, with *F* set to zero for negative *F*^2^. The threshold expression of *F*^2^ > σ(*F*^2^) is used only for calculating *R*-factors(gt) *etc*. and is not relevant to the choice of reflections for refinement. *R*-factors based on *F*^2^ are statistically about twice as large as those based on *F*, and *R*- factors based on ALL data will be even larger.

### Fractional atomic coordinates and isotropic or equivalent isotropic displacement parameters (Å^2^)

**Table:**

	*x*	*y*	*z*	*U*_iso_*/*U*_eq_	
Cl1	1.07800 (5)	0.08403 (3)	−0.18284 (3)	0.03779 (9)	
S1	0.17791 (4)	0.27870 (3)	0.20939 (2)	0.02534 (8)	
O1	−0.27472 (13)	0.52923 (9)	0.54724 (7)	0.02723 (18)	
O2	−0.14178 (14)	0.66439 (9)	0.43584 (8)	0.02957 (19)	
N1	0.32516 (14)	0.31817 (9)	0.01686 (8)	0.02134 (17)	
N2	0.16931 (14)	0.38648 (10)	0.02374 (7)	0.02176 (17)	
N3	−0.07075 (13)	0.42674 (10)	0.12269 (8)	0.02133 (17)	
N4	−0.14591 (13)	0.45733 (9)	0.21809 (7)	0.01985 (17)	
N5	−0.27251 (13)	0.33810 (9)	0.45894 (7)	0.01958 (16)	
N6	−0.32110 (15)	0.39514 (10)	0.55021 (8)	0.02548 (19)	
C1	0.63437 (17)	0.17276 (12)	−0.03615 (9)	0.0237 (2)	
H1A	0.5690	0.1433	0.0185	0.028*	
C2	0.78848 (17)	0.11197 (12)	−0.06418 (10)	0.0265 (2)	
H2A	0.8263	0.0413	−0.0295	0.032*	
C3	0.88538 (17)	0.15913 (12)	−0.14545 (10)	0.0264 (2)	
C4	0.83211 (18)	0.26371 (12)	−0.19913 (10)	0.0275 (2)	
H4A	0.8994	0.2941	−0.2527	0.033*	
C5	0.67620 (18)	0.32191 (11)	−0.17115 (9)	0.0250 (2)	
H5A	0.6374	0.3911	−0.2073	0.030*	
C6	0.57626 (16)	0.27820 (11)	−0.08939 (9)	0.02146 (19)	
C7	0.41298 (16)	0.34357 (11)	−0.06406 (9)	0.0225 (2)	
H7A	0.3715	0.4053	−0.1077	0.027*	
C8	0.08764 (15)	0.36783 (10)	0.10979 (8)	0.01926 (18)	
C9	−0.02848 (18)	0.25579 (12)	0.27750 (11)	0.0272 (2)	
H9A	−0.1132	0.1856	0.2353	0.033*	
H9B	0.0039	0.2276	0.3479	0.033*	
C10	−0.12334 (14)	0.38080 (10)	0.29279 (9)	0.01908 (18)	
C11	−0.19794 (15)	0.42203 (10)	0.39393 (9)	0.01953 (18)	
C12	−0.19652 (16)	0.55221 (11)	0.45021 (9)	0.0229 (2)	
C13	−0.30832 (14)	0.19503 (10)	0.43535 (9)	0.01922 (18)	
C14	−0.22133 (15)	0.11515 (11)	0.50272 (9)	0.02130 (19)	
H14A	−0.1436	0.1523	0.5641	0.026*	
C15	−0.25405 (16)	−0.02255 (11)	0.47558 (10)	0.0241 (2)	
H15A	−0.1976	−0.0784	0.5194	0.029*	
C16	−0.36970 (17)	−0.07694 (12)	0.38406 (11)	0.0257 (2)	
H16A	−0.3901	−0.1690	0.3667	0.031*	
C17	−0.45555 (17)	0.00539 (12)	0.31792 (10)	0.0261 (2)	
H17A	−0.5334	−0.0318	0.2566	0.031*	
C18	−0.42526 (15)	0.14293 (12)	0.34325 (10)	0.0235 (2)	
H18A	−0.4820	0.1988	0.2995	0.028*	
H1N3	−0.095 (3)	0.481 (2)	0.0777 (16)	0.040 (5)*	

### Atomic displacement parameters (Å^2^)

**Table:**

	*U*^11^	*U*^22^	*U*^33^	*U*^12^	*U*^13^	*U*^23^
Cl1	0.02970 (16)	0.03100 (16)	0.0513 (2)	0.00136 (11)	0.01490 (14)	−0.00771 (13)
S1	0.02671 (14)	0.02899 (15)	0.02507 (14)	0.01210 (10)	0.00651 (10)	0.01304 (10)
O1	0.0348 (5)	0.0217 (4)	0.0254 (4)	0.0064 (3)	0.0034 (3)	0.0011 (3)
O2	0.0342 (5)	0.0175 (4)	0.0367 (5)	0.0035 (3)	0.0001 (4)	0.0044 (3)
N1	0.0247 (4)	0.0201 (4)	0.0196 (4)	0.0045 (3)	0.0019 (3)	0.0024 (3)
N2	0.0257 (4)	0.0222 (4)	0.0183 (4)	0.0059 (3)	0.0016 (3)	0.0039 (3)
N3	0.0241 (4)	0.0227 (4)	0.0187 (4)	0.0064 (3)	0.0012 (3)	0.0067 (3)
N4	0.0212 (4)	0.0194 (4)	0.0196 (4)	0.0031 (3)	0.0011 (3)	0.0054 (3)
N5	0.0213 (4)	0.0185 (4)	0.0200 (4)	0.0044 (3)	0.0019 (3)	0.0051 (3)
N6	0.0314 (5)	0.0235 (4)	0.0228 (4)	0.0057 (4)	0.0055 (4)	0.0042 (3)
C1	0.0275 (5)	0.0233 (5)	0.0211 (5)	0.0033 (4)	0.0040 (4)	0.0037 (4)
C2	0.0280 (5)	0.0254 (5)	0.0262 (5)	0.0047 (4)	0.0037 (4)	0.0021 (4)
C3	0.0251 (5)	0.0236 (5)	0.0285 (5)	−0.0011 (4)	0.0061 (4)	−0.0051 (4)
C4	0.0320 (6)	0.0228 (5)	0.0259 (5)	−0.0057 (4)	0.0093 (4)	−0.0023 (4)
C5	0.0336 (6)	0.0194 (5)	0.0213 (5)	−0.0027 (4)	0.0054 (4)	0.0014 (4)
C6	0.0261 (5)	0.0193 (4)	0.0184 (4)	0.0010 (4)	0.0027 (4)	0.0007 (3)
C7	0.0286 (5)	0.0199 (5)	0.0192 (4)	0.0036 (4)	0.0022 (4)	0.0028 (3)
C8	0.0220 (4)	0.0168 (4)	0.0187 (4)	0.0023 (3)	−0.0007 (3)	0.0032 (3)
C9	0.0342 (6)	0.0197 (5)	0.0329 (6)	0.0097 (4)	0.0137 (5)	0.0118 (4)
C10	0.0200 (4)	0.0160 (4)	0.0223 (4)	0.0030 (3)	0.0022 (3)	0.0058 (3)
C11	0.0214 (4)	0.0167 (4)	0.0216 (4)	0.0038 (3)	0.0014 (3)	0.0059 (3)
C12	0.0240 (5)	0.0197 (5)	0.0251 (5)	0.0053 (4)	−0.0007 (4)	0.0040 (4)
C13	0.0198 (4)	0.0168 (4)	0.0222 (4)	0.0021 (3)	0.0038 (3)	0.0057 (3)
C14	0.0233 (5)	0.0209 (5)	0.0213 (5)	0.0043 (4)	0.0035 (4)	0.0070 (3)
C15	0.0247 (5)	0.0205 (5)	0.0301 (5)	0.0059 (4)	0.0074 (4)	0.0091 (4)
C16	0.0247 (5)	0.0189 (5)	0.0347 (6)	0.0008 (4)	0.0099 (4)	0.0039 (4)
C17	0.0227 (5)	0.0252 (5)	0.0290 (5)	−0.0025 (4)	0.0019 (4)	0.0018 (4)
C18	0.0205 (5)	0.0242 (5)	0.0259 (5)	0.0004 (4)	−0.0005 (4)	0.0074 (4)

### Geometric parameters (Å, °)

**Table:**

Cl1—C3	1.7379 (13)	C4—C5	1.3861 (18)
S1—C8	1.7374 (10)	C4—H4A	0.9300
S1—C9	1.8097 (12)	C5—C6	1.3984 (16)
O1—N6	1.3788 (13)	C5—H5A	0.9300
O1—C12	1.4197 (15)	C6—C7	1.4607 (16)
O2—C12	1.2117 (14)	C7—H7A	0.9300
N1—C7	1.2837 (15)	C9—C10	1.5030 (15)
N1—N2	1.3921 (13)	C9—H9A	0.9700
N2—C8	1.3000 (14)	C9—H9B	0.9700
N3—C8	1.3659 (14)	C10—C11	1.4526 (15)
N3—N4	1.3712 (13)	C11—C12	1.4217 (15)
N3—H1N3	0.85 (2)	C13—C14	1.3870 (14)
N4—C10	1.2896 (13)	C13—C18	1.3881 (16)
N5—N6	1.3101 (14)	C14—C15	1.3935 (16)
N5—C11	1.3563 (13)	C14—H14A	0.9300
N5—C13	1.4434 (14)	C15—C16	1.3836 (18)
C1—C2	1.3874 (17)	C15—H15A	0.9300
C1—C6	1.4018 (16)	C16—C17	1.3912 (18)
C1—H1A	0.9300	C16—H16A	0.9300
C2—C3	1.3940 (17)	C17—C18	1.3886 (17)
C2—H2A	0.9300	C17—H17A	0.9300
C3—C4	1.3861 (19)	C18—H18A	0.9300
			
C8—S1—C9	97.34 (5)	N3—C8—S1	119.98 (8)
N6—O1—C12	111.31 (8)	C10—C9—S1	112.38 (8)
C7—N1—N2	112.59 (9)	C10—C9—H9A	109.1
C8—N2—N1	112.52 (9)	S1—C9—H9A	109.1
C8—N3—N4	126.88 (9)	C10—C9—H9B	109.1
C8—N3—H1N3	114.7 (14)	S1—C9—H9B	109.1
N4—N3—H1N3	111.8 (14)	H9A—C9—H9B	107.9
C10—N4—N3	118.20 (9)	N4—C10—C11	115.66 (9)
N6—N5—C11	115.55 (9)	N4—C10—C9	123.01 (10)
N6—N5—C13	117.95 (9)	C11—C10—C9	121.33 (9)
C11—N5—C13	126.46 (9)	N5—C11—C12	105.58 (9)
N5—N6—O1	104.02 (9)	N5—C11—C10	125.07 (9)
C2—C1—C6	120.65 (11)	C12—C11—C10	129.17 (10)
C2—C1—H1A	119.7	O2—C12—O1	120.14 (11)
C6—C1—H1A	119.7	O2—C12—C11	136.24 (12)
C1—C2—C3	118.54 (11)	O1—C12—C11	103.54 (9)
C1—C2—H2A	120.7	C14—C13—C18	122.57 (10)
C3—C2—H2A	120.7	C14—C13—N5	119.37 (10)
C4—C3—C2	122.18 (11)	C18—C13—N5	118.04 (9)
C4—C3—Cl1	118.32 (10)	C13—C14—C15	117.91 (10)
C2—C3—Cl1	119.49 (10)	C13—C14—H14A	121.0
C5—C4—C3	118.46 (11)	C15—C14—H14A	121.0
C5—C4—H4A	120.8	C16—C15—C14	120.63 (10)
C3—C4—H4A	120.8	C16—C15—H15A	119.7
C4—C5—C6	121.04 (11)	C14—C15—H15A	119.7
C4—C5—H5A	119.5	C15—C16—C17	120.33 (11)
C6—C5—H5A	119.5	C15—C16—H16A	119.8
C5—C6—C1	119.11 (11)	C17—C16—H16A	119.8
C5—C6—C7	118.29 (10)	C18—C17—C16	120.13 (11)
C1—C6—C7	122.59 (10)	C18—C17—H17A	119.9
N1—C7—C6	121.78 (10)	C16—C17—H17A	119.9
N1—C7—H7A	119.1	C13—C18—C17	118.43 (10)
C6—C7—H7A	119.1	C13—C18—H18A	120.8
N2—C8—N3	117.39 (9)	C17—C18—H18A	120.8
N2—C8—S1	122.56 (8)		
			
C7—N1—N2—C8	−175.59 (10)	S1—C9—C10—C11	−137.68 (9)
C8—N3—N4—C10	−32.21 (16)	N6—N5—C11—C12	0.24 (13)
C11—N5—N6—O1	−0.32 (13)	C13—N5—C11—C12	177.95 (10)
C13—N5—N6—O1	−178.24 (9)	N6—N5—C11—C10	175.72 (10)
C12—O1—N6—N5	0.28 (12)	C13—N5—C11—C10	−6.57 (17)
C6—C1—C2—C3	0.77 (18)	N4—C10—C11—N5	145.80 (11)
C1—C2—C3—C4	−0.37 (19)	C9—C10—C11—N5	−33.52 (17)
C1—C2—C3—Cl1	−179.55 (9)	N4—C10—C11—C12	−39.82 (16)
C2—C3—C4—C5	−0.58 (18)	C9—C10—C11—C12	140.87 (12)
Cl1—C3—C4—C5	178.61 (9)	N6—O1—C12—O2	−177.42 (10)
C3—C4—C5—C6	1.14 (18)	N6—O1—C12—C11	−0.15 (12)
C4—C5—C6—C1	−0.75 (17)	N5—C11—C12—O2	176.55 (13)
C4—C5—C6—C7	−179.85 (11)	C10—C11—C12—O2	1.3 (2)
C2—C1—C6—C5	−0.23 (17)	N5—C11—C12—O1	−0.04 (11)
C2—C1—C6—C7	178.83 (11)	C10—C11—C12—O1	−175.28 (10)
N2—N1—C7—C6	−177.30 (10)	N6—N5—C13—C14	−63.26 (14)
C5—C6—C7—N1	−172.94 (11)	C11—N5—C13—C14	119.08 (12)
C1—C6—C7—N1	8.00 (18)	N6—N5—C13—C18	118.73 (11)
N1—N2—C8—N3	−177.81 (9)	C11—N5—C13—C18	−58.93 (15)
N1—N2—C8—S1	5.18 (14)	C18—C13—C14—C15	0.06 (16)
N4—N3—C8—N2	−157.47 (11)	N5—C13—C14—C15	−177.86 (10)
N4—N3—C8—S1	19.62 (15)	C13—C14—C15—C16	0.09 (16)
C9—S1—C8—N2	−165.26 (10)	C14—C15—C16—C17	−0.21 (17)
C9—S1—C8—N3	17.80 (10)	C15—C16—C17—C18	0.17 (18)
C8—S1—C9—C10	−43.84 (10)	C14—C13—C18—C17	−0.10 (17)
N3—N4—C10—C11	177.00 (9)	N5—C13—C18—C17	177.85 (10)
N3—N4—C10—C9	−3.70 (16)	C16—C17—C18—C13	−0.01 (17)
S1—C9—C10—N4	43.07 (15)		

### Hydrogen-bond geometry (Å, °)

**Table:**

Cg2 is the centroid of the C1–C6 benzene ring.

**Table:**

*D*—H···*A*	*D*—H	H···*A*	*D*···*A*	*D*—H···*A*
N3—H1N3···N2^i^	0.84 (2)	2.03 (2)	2.8752 (14)	178 (2)
C9—H9A···Cl1^ii^	0.97	2.78	3.4904 (13)	130
C18—H18A···S1^iii^	0.93	2.86	3.6729 (12)	147
C17—H17A···Cg2^iv^	0.93	2.64	3.5208 (15)	158

Symmetry codes: (i) −*x*, −*y*+1, −*z*; (ii) −*x*+1, −*y*, −*z*; (iii) *x*−1, *y*, *z*; (iv) −*x*, −*y*, −*z*.
